# Influence of Particle Size and Zeta Potential in Treating Highly Coloured Old Landfill Leachate by Tin Tetrachloride and Rubber Seed

**DOI:** 10.3390/ijerph19053016

**Published:** 2022-03-04

**Authors:** Siti Fatihah Ramli, Hamidi Abdul Aziz, Fatehah Mohd Omar, Mohd Suffian Yusoff, Herni Halim, Mohamad Anuar Kamaruddin, Kamar Shah Ariffin, Yung-Tse Hung

**Affiliations:** 1 School of Civil Engineering, Engineering Campus, Universiti Sains Malaysia, Seberang Perai 14300, Malaysia; ctfatihah88@gmail.com (S.F.R.); cefatehah@usm.my (F.M.O.); suffian@usm.my (M.S.Y.); ceherni@usm.my (H.H.); 2 Solid Waste Management Cluster, Science and Technology Research Centre, Engineering Campus, Universiti Sains Malaysia, Seberang Perai 14300, Malaysia; 3 School of Industrial Technology, Universiti Sains Malaysia, Gelugor 11800, Malaysia; anuarkamaruddin@usm.my; 4 School of Materials and Mineral Resources Engineering, Engineering Campus, Universiti Sains Malaysia, Seberang Perai 14300, Malaysia; kamarsha@usm.my; 5 Department of Civil and Environmental Engineering, Cleveland State University, Cleveland, OH 44115, USA; yungtsehung@gmail.com

**Keywords:** solid waste, landfill, leachate, tin tetrachloride, *Hevea brasiliensis*

## Abstract

Old leachate normally has a low organic compound content, poor biodegradability and is hard to biologically treat. The efficacy of tetravalent metal salts as a coagulant and the application of a natural coagulant as a flocculant in landfill leachate treatment is still inconclusive. Hence, this study aimed to evaluate the potential application of tin tetrachloride (SnCl_4_) as the main coagulant and the rubber seed (*Hevea brasiliensis*) (RS) as the natural coagulant aid as the sole treatment in eradicating highly coloured and turbid stabilised landfill leachate present at one of the old local landfills in Malaysia. The standard jar test conducted revealed that SnCl_4_ was able to eliminate 99% and 97.3% of suspended solids (SS) and colour, respectively, at pH8, with 10,000 mg/L dosages, an average particle size of 2419 d·nm, and a zeta potential (ZP) of −0.4 mV. However, RS was found to be ineffective as the main coagulant and could only remove 46.7% of SS and 76.5% of colour at pH3 with 6000 mg/L dosages, and also exhibited smaller particles (933 d·nm) with ZP values of −6.3 mV. When used as a coagulant aid, the polymer bridging mechanism in RS helped in reducing the SnCl_4_ concentration from 10,000 mg/L to 8000 mg/L by maintaining the same performances. The presence of 1000 mg/L RS as a coagulant aid was able to remove 100% of SS and 97.6% of colour. The study concluded that RS has the potential to be used together with SnCl_4_ in treating concentrated leachate with SS and colour.

## 1. Introduction

Among the available methods of waste disposal, landfilling is still one of the most commonly used methods worldwide. This is because compared to the other waste disposal methods, landfilling provides a more economical and simple procedure to be applied. However, without proper management, it may lead to serious environmental problems, as landfilling tends to produce toxic gases and liquids, such as leachate [1,2]. Leachate is high-strength wastewater and is black in colour, containing numerous toxic pollutants that can pose a danger to the surroundings. Old leachate is categorised as stabilised and matured leachate, which is in the methanogenic phase. Stabilized leachate usually contains a lower concentration of organic compounds than younger leachate [3]. However, this leachate has low biodegradability due to its high content of non-biodegradable organic compounds [4,5,6]. Physical-chemical processes such as the coagulation-flocculation technique are appropriate and recommended for stabilised leachate to treat chemical oxygen demand (COD), suspended solids (SS), colour, and heavy metals [7,8]. Leachate is very difficult to deal with and to treat due to its complex composition. It normally requires multiple treatment methods, which depend on the type and age of the landfill.

The process of removing the pollutants from the leachate can be achieved by disrupting the stability of its surface particles. A coagulation and flocculation procedure causes the surface charge of the particles to be reduced or destabilised by the addition of chemicals, known as a coagulant. Meanwhile, the flocculation process involves the agglomeration of particles, forming into a bigger size [9,10,11]. By applying the coagulation and flocculation treatments, concentrated heavy metals, organics, colour, chemical oxygen demand (COD) and total suspended solids are expected to be removed effectively [12].

Primary coagulants and coagulant aids are used in this process. Usually, primary coagulants consist of metal salts, and coagulant aids are mostly derived from polymers/polyelectrolytes. The coagulant aid acts to improve the roughness of the floc, hence increasing its density, which then minimises the breakup of the floc during the mixing and settling process, as well as reducing the flocculation and settling period [13,14]. The amount of primary coagulant (metal salt coagulant) in the treatment process can be decreased by the coagulant aid. At the same time, the sludge also contains a lower level of chemical residues.

The amount of electrolyte needed to coagulate a certain quantity of a colloidal solution relies on the valency of the ion having a counter charge to that of the colloidal particles. As stated by the Schulze-Hardy rule, stability decreases with the increase in counter-ion valency [15]. The degree of destabilisation is different depending on the coagulant. Coagulant with a higher valence of the counter-ion provides a greater destabilisation effect, which in turn lowers the dose required in the process [16]. This is due to the attractive electrostatic forces between the ions. The ionic strength of a metal ion (of a salt) used in the destabilisation of the colloidal particles is determined by the valence. As the electrostatic forces of repulsion are decreased, the attraction forces of van der Waals are increased, which induce the ions’ kinetic movement through the diffuse layer [17]. Hence, for the coagulation of negatively charged particles, tetravalent cations are more efficient than trivalent cations, which are more efficient than divalent ions, which are far more efficient than monovalent cations.

Several works have been performed to identify the possibility of four valence state coagulants through the coagulation-flocculation process. Zhao et al., (2013) [18] used the poly titanium tetrachloride (PTC) in treating synthetic water, which removed 89.2% of UV_254_ and 61.6% of dissolved organic carbon (DOC). Aziz et al., (2015) [19] studied leachate treatment by titanium tetrachloride (TiCl_4_) obtained 81.4%, 86.7%, and 76.5% reductions in colour, SS, and ammoniacal nitrogen, respectively. At the same time, Aziz and Ramli (2014) [20] found that zirconium tetrachloride (ZrCl_4_) was able to remove 93.4% of SS, 94.3% of colour and 97% of UV_254_ from the leachate. We used tin (IV) chloride (SnCl_4_) as a sole coagulant in our previous works in treating COD in the same leachate as in the current study. The optimum conditions occurred at pH7.17 and SnCl_4_ dosage of 15,012 mg/L, which exhibited 67.6% reductions in COD [21].

Tin salts have been applied in most other water/wastewater treatments. For example, a study conducted by Mathews et al., (2015) [22] involves the use of tin (II) chloride in the removal of Hg (mercury) in stream water by air stripping method. From the study, it was found that the use of SnCl_2_ as the coagulant was able to remove more than 90% of Hg (mercury) from the stream water. The removal achieved was relatively higher. In addition, another study conducted by Kennedy et al., (2020) [23] found that the use of SnCl_2_ together with rapid sand filtration in a pilot scale in the treatment of groundwater achieved a consistent removal of chromium (Cr) from the groundwater. Moreover, Zepeda et al., (2018) [24] applied the usage of tin oxide (SnO_2_) synthesised from SnCl_4_, finding that it allowed the significant removal of both nickel (Ni^2+^) and copper (Cu^2+^) ions from an aqueous solution. Therefore, theoretically, it was expected that SnCl_4_, as the tetravalent coagulant with four valencies, is able to remove and act as one of the best coagulants in removing pollutants from the leachate.

Hence, the current work was undertaken to examine the possibility of employing tin (IV) chloride and RS as an alternative coagulant and coagulant aid (flocculant), respectively. These substances have not yet been tested together in treating water, wastewater, and landfill leachate as a sole treatment. The target pollutant parameters were colour and SS, which are among the hardest parameters to treat in stabilised leachate [25]. The goal of this research was to assess the possibility of reducing the primary coagulant quantity of tin (IV) chloride (SnCl_4_) by adding RS as a flocculant.

## 2. Materials and Methods

### 2.1. Characterisation of Leachate

The case study leachate was sampled from the Alor Pongsu Landfill Site (APLS), located at 5°04′ N, 100°35′ E in Alor Pongsu, Perak, Malaysia. The monitoring of the leachate characteristics was conducted for 12 months starting from November 2018 to October 2019, and the results are outlined in Table 1.

The BOD_5_/COD ratio of Alor Pongsu leachate is 0.03, which is <0.1; hence, it is in a stable form and is difficult to biologically treat. It is necessary for this type of leachate to be pretreated so that the organic compound becomes much simpler and can easily be broken down by the bacteria. The colour level is also considered extremely high (maximum value almost reached 23,000 PtCo), and this could be associated with the high organic content (humic and fulvic acids) present in the leachate.

### 2.2. Preparation of Tin Tetrachloride (SnCl_4_) Stock Solution

Tin tetrachloride (SnCl_4_) was provided by BG OilChem Sdn. Bhd. (Permatang Pauh, Pulau Pinang, Malaysia). The stock solution of 40 g/L was prepared, and the appropriate working concentrations were obtained based on the dilution principle. The working concentrations of SnCl_4_ varied from 8000 mg/L to 18,000 mg/L.

### 2.3. Preparation of Rubber Seed (Hevea brasiliensis) Stock Solution

The raw rubber seeds used in this study were obtained from a local rubber tree farm located in Sg. Petani, Kedah, Malaysia. The seed coats were removed, and the seeds were dried at 105 °C in an oven for 30–60 min to remove the moisture. Next, a heavy-duty grinder was used for crushing the dried rubber seeds into powder. The stock solution of rubber seed was prepared by mixing the rubber seed powder with distilled water, and the mixture was agitated at room temperature for 30 min. Then, the blend was filtered thrice using a filter cloth. The solution was then diluted to the desired concentration needed for the experiment. The working concentrations used for RS were varied from 1000 mg/L to 10,000 mg/L. These were prepared by adding an appropriate amount of RS into distilled water. One gram of RS added into 1 L of distilled water is equivalent to a 1000 mg/L solution. The non-diluted solution was made daily and used on the day it was prepared.

### 2.4. Zeta Potential and Particle Size Measurement

The zeta potential was evaluated using a 25 °C Malvern Zetasizer Nano ZS (Malvern Instruments, Malvern, UK) instrument. The measurement was conducted for the raw leachate and also for the sample obtained immediately after the coagulation and flocculation process. All samples were tested in triplicate. For each test, the capillary cell was injected with 1 mL of supernatant by means of a syringe [26]. The particle electrophoretic mobility was measured by means of the light dispersion/dynamic light scattering method. This method is sometimes also referred to as photon correlation spectroscopy or quasi-elastic light scattering, and is a technique used for measuring the size or particles especially in the sub-micron region. The sample was tested following the standard procedures provided in the Malvern Zetasizer manual. In an applied electric field, the motion of the particles leads to a minor frequency change in the dispersed light, and laser Doppler electrophoresis typically detects this slight movement [27,28].

### 2.5. Coagulation and Flocculation Test

The coagulation and flocculation tests were conducted in the laboratory by means of a standard jar test instrument equipped with 6 paddles (VELP Scientifica Srl, Usmate, Italy). The leachate samples used in the experiment was 500 mL for each beaker. Therefore, a total of 3 L was required for the 6 beakers. To prevent the settling of solids, the leachate sample was completely agitated, and the sample was allowed to condition at the ambient temperature for about 30 min. Using 3 M hydrochloric acid (HCl) or 3 M sodium hydroxide, the pH of the samples was modified to the appropriate pH value (pH3–11) (NaOH). The acid and base used in this study were also used by other researchers, for example [29,30]. Then, using the pre-determined value of the operational condition, the leachate was agitated simultaneously. For the determination of SnCl_4_ and RS as the main coagulant, the doses were varied from 8000 mg/L to 18,000 mg/L and from 1000 mg/L to 10,000 mg/L, respectively. Meanwhile, for the determination of RS as the coagulant aid, the doses were varied in the range of 100 mg/L to 250 mg/L, 500 mg/L, 750 mg/L, 1000 mg/L, 2000 mg/L, 4000 mg/L, 6000 mg/L, 8000 mg/L and 10,000 mg/L. The tests were conducted at an optimum pH8. The jar test was run at 220 rpm for 5 min (rapid mixing) and 60 rpm for 30 min (slow mixing). The samples were allowed to settle for 40 min. The samples were then tested for zeta potential, colour and suspended solids. The removal percentages were calculated based on the SS and colour concentrations before (raw) and after the experiments in relation to their raw concentrations.

Percentage Removal (%) = [(*C_i_* − *C_f_*)/*C_i_*] × 100 (1)

where:

*C_i_* = initial concentrations.

*C_f_* = final of suspended solids and colour.

## 3. Results and Discussion

### 3.1. Influence of Coagulant Dosages

**Figure 1:**
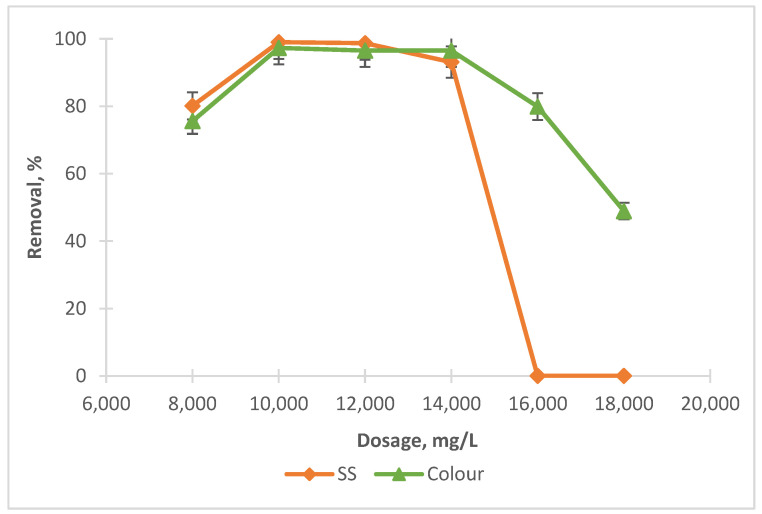
Impact of coagulant dosage on SS and colour removals with SnCl_4_.

**Figure 2:**
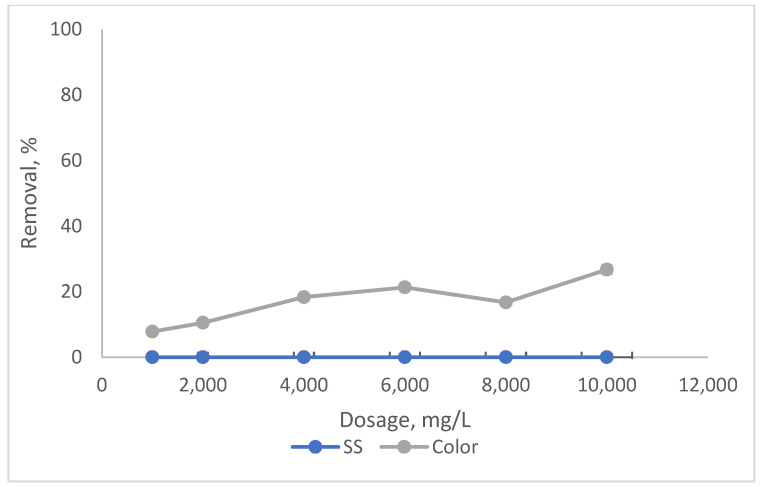
Impact of coagulant dosage on SS and colour removals with RS.

Figure 1 and Figure 2 illustrate the effect of tin tetrachloride (SnCl_4_) and rubber seed (RS) dosage on the removal performance. The tests were conducted by varying the coagulant dosage of SnCl_4_ and RS at leachate original pH, which is 8.24. Based on the preliminary experiments, for SnCl_4_, the dosages were varied from 8000 mg/L to 18,000 mg/L, while for RS, the dosages of coagulants ranged from 1000 mg/L to 10,000 mg/L.

It can be noted from Figure 1 that when the amount of SnCl_4_ was increased from 8000 mg/L to 10,000 mg/L, the elimination for SS and colour also increased from 80.1% to 99% and 75.6% to 97.3%, respectively. Beyond 10,000 mg/L, the removals for both dropped, gradually for colour and significantly for SS. This was due to the overdosing of coagulant dosage, which leads to the destabilisation of the particles (partly associated as organics in colour) and hence gives a lower removal compared to at the optimum point [31]. However, RS was found not as effective as a coagulant, as shown in Figure 2. At an optimal dosage of 6000 mg/L, the removals for both were less than 30%, a little bit better for colour (about 23%) compared to SS. This is because the addition of RS tends to increase the organic matter content in leachate, which could not be effectively settled even after the treatment.

### 3.2. Influence of pH

**Figure 3:**
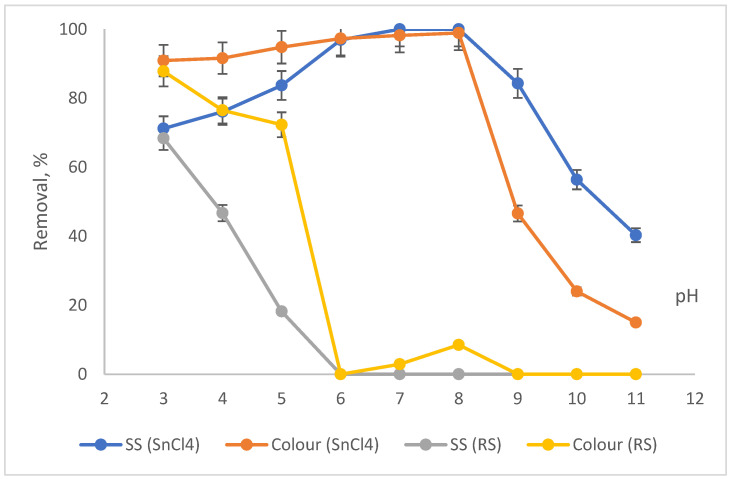
Impact of pH on SS and colour removals by SnCl_4_ and RSS.

The impact of pH on performance is shown in Figure 3. The pH values were varied from pH3 to pH11. We excluded pH2 and pH12 because these values are too extreme. The optimum pre-determined dosages for both SnCl_4_ (10,000 mg/L) and RS (6000 mg/L) were applied in this investigation.

Based on Figure 3, the removal of SS and colour increased with the increase in pH from 3 to pH8, when SnCl_4_ was applied as the main coagulant. The elimination was increased from 71.2% for SS and 90.9% for colour at pH3 to almost complete removal (100%) at pH8 for both. Beyond pH8 as the optimum, the removals gradually dropped until pH11, where the removals for SS and colour were 40.3% for colour and 15% for SS. A number of soluble hydrolysis species were then produced when SnCl_4_ (a metal salt coagulant) was applied to the sample. The species may be charged either positively or negatively, based on the pH of the sample. The formation of a positively charged surface could destabilise the negatively charged colloidal particles. The destabilisation of the particles will lead to the aggregation of particles and the establishment of flocs. The condition where positively charged surface species adsorb onto the surface of the negatively charged particles is called a charged neutralisation mechanism [32]. When the amount of the coagulant exceeds the optimum level, this overdosing condition leads to the destabilisation of the particles, which hinders the settlement. As the SS also constitutes some organics that may also contribute to the colour level, a non-efficient settlement of particles would also exhibit poor removal of colour.

As opposed to the SnCl_4_, when RS was applied as the main coagulant, the SS and colour reductions decreased with the increase in the pH values. The highest removal was at pH3 with 68.4% and 87.8% removals for SS and colour, respectively. Then, as the pH increased from pH4 to 11, the reduction in SS and colour started to gradually drop until it reached the lowest removal at pH 6 with less than 5% reductions for both. Better removals at pH3 are due to the acidic effect from the acid used and not due to the RS applied. A few studies on natural coagulant have also reported good performances at acidic pH. Kristianto (2017) [33] used *Leucaena leucocephala* seed’s extract as a natural coagulant to eradicate dye from synthetic wastewater and managed to reduce 99% of colour at pH3. In addition, Zonoozi et al., (2011) [34] removed 83% of colour from dye-containing solutions when chitosan was employed as the main natural coagulant. The high performance obtained at acidic conditions is due to the protonation of the extracted protein from the natural coagulant. Basically, under acidic conditions, a higher H^+^ concentration from the protonation will neutralise the negative charges of the wastewater surface particles. Therefore, more removal was obtained under this condition when natural coagulant was used as the main coagulant [20,33,34].

### 3.3. Rubber Seed as Coagulant Aid

**Figure 4:**
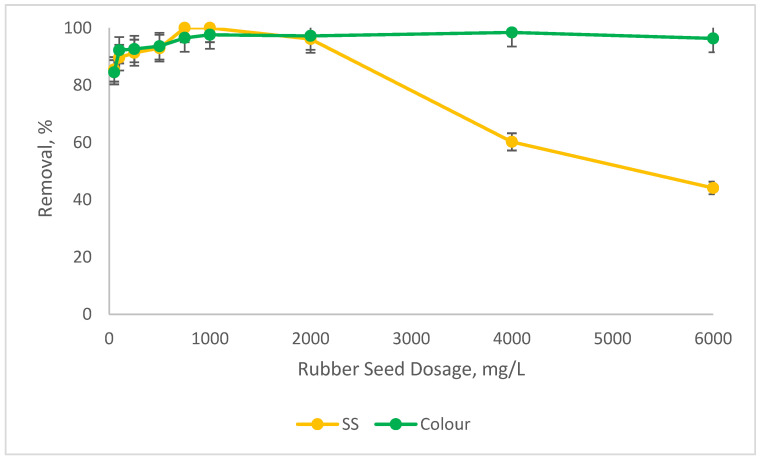
Percentage reductions in SS and colour at 8000 mg/L SnCl_4_ with different ranges of RS as the coagulant aid at pH8.

We examined the potential of RS as a coagulant aid in reducing the optimum SnCl_4_ dose (10,000 mg/L) as determined in the previous experiment. We ranged the SnCl_4_ dosages from 4000 mg/L to 8000 mg/L. Our previous experiment indicated that the SnCl_4_ dosage could not be reduced below 8000 mg/L, as the removal for both SS and colour were fairly low (less than 50%), as also shown in Figure 1. This is because too much or not enough coagulant added into water/wastewater will make the removal less efficient. This is due to the overdosing or insufficient surface charge to affect the stability of the colloid’s particles [35]. For this, we run the experiment at a fixed SnCl_4_ of 8000 mg/L, and we vary the coagulant aid dosages (RS) from 100 mg/L to 250 mg/L, 500 mg/L, 750 mg/L, 1000 mg/L, 2000 mg/L, 4000 mg/L, 6000 mg/L, 8000 mg/L and 10,000 mg/L. The tests were undertaken at an optimum pH8. The results are shown in Figure 4.

Figure 4 indicates that the removal of SS and colour increased as the RS dosages increased. When the RS dose rose from 100 mg/L to 1000 mg/L, the removal for SS was improved from about 90% to almost 100% and from 92% to about 97% for colour. Beyond 1000 mg/L, the reductions in the performance for colour were not significant and were maintained above 90% at different RS dosages. However, there is a significant drop in the SS removals beyond 1000 mg/L of RS. This is due to the overdosing phenomenon, as explained before. Even though the concentration of SnCl_4_ was reduced from 10,000 mg/L to 8000 mg/L, in the presence of RS as a coagulant aid, the removals for SS and colour were on par with the performance of SnCl_4_ alone, as presented in Figure 1. According to Aygun and Yilmaz (2010) [32], the use of natural polyelectrolyte as coagulant aid can assist in reducing the concentration of metal coagulant without affecting the removal of the parameters. Basically, there are four types of mechanisms involved in the coagulation and flocculation process. In the case of natural coagulant aid, the possible mechanism is charge neutralisation or polymer bridging. However, compared to charge neutralisation, the polymer bridging mechanism is more dominant in plant-based coagulant aid. During the polymer bridging mechanism, the surface of the colloidal particles will attach to the polymer chains because of the existence of affinity between them. In addition, for a bridging mechanism to be effective, the addition of enough polymer dosage is an important criterion, as it helps to provide a sufficient site for particle attachment. It will provide sufficient unoccupied surface for particle attachment and the bridging span, which should be at a distance so that the interparticle repulsion between the particles is hindered. Therefore, adequate natural coagulant dosage will provide sufficient bridging links and surfaces for the removal of pollutants [36]. In polymer bridging, the particle surface of the colloid is able to attach to the loop of RS polymer chain. This polymer bridging mechanism helps in reducing the amount of chemical dosage (SnCl_4_), through the charge neutralisation mechanism. The combination of these two mechanisms is able to remove a high percentage of SS and colour.

Organic matter (some in insoluble forms), measured as turbidity, and SS contribute mainly to colour in landfill leachates [13]. SnCl_4_ is a cationic coagulant with positive electric charges. It helps to decrease the negative charge of hydroxide-forming colloids when dissolved in water. This hydroxide is hydrophobic in nature, meaning it can adsorb to the surface of organic anionic particles and become insoluble [37]. According to (Awang et al., 2012) [38], ‘bridging’ is the main mechanism that governs the agglomeration of the constituents for the coagulants that present as a negative surface charge. The addition of RS could help to form bigger flocs and enhance particle settlement, thus improving the removal rates. According to the findings, it was found that RS could be used as a coagulant aid to minimise the amount of metal coagulant used.

### 3.4. Comparisons of Zeta Potential (ZP) and Particle Size of SnCl_4_ and Rubber Seed

**Figure 5:**
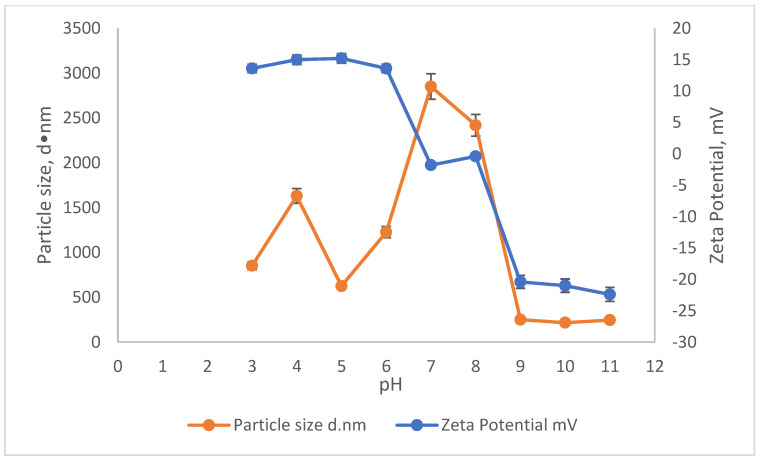
The influence of pH on the Zeta potential and particle size of SnCl_4_.

**Figure 6:**
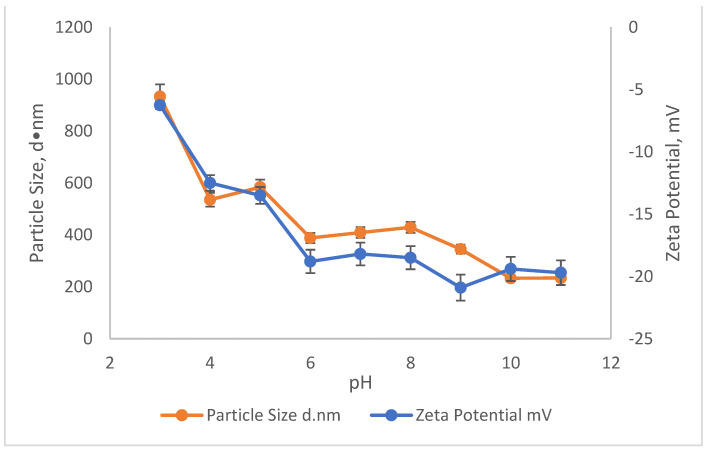
The influence of pH on the Zeta potential and Particle Size of Rubber Seed.

The effect of pH on the ZP and particle size on the reduction in SS and colour using SnCl_4_ and RS are illustrated in Figure 5 and Figure 6. The particle size measurement was carried out by using the Malvern Zetasizer Nano ZS by applying the dynamic light scattering method (DLS). First, a laser is employed to provide a light source for the sample particles within a cell. Then, the intensity of the dispersed light is measured using a detector. Because a particle scatters light in all directions, the detector can theoretically be placed anywhere and yet detect the scattering. The detector’s scattering intensity signal is sent to a correlator, which is a digital signal processor. The information from the correlator is then sent to a computer, where the Zetasizer software analyses the data and calculates the size. The determination of the sample particle size in this study is important, as it provides the effectiveness of the coagulant when added into the treatment. This is because, depending on the surface charge of the coagulant, the agglomeration will be induced to form a bigger size of particles/colloid, which then settles down during the settlement process. The relationship of ZP and particle size in removing the SS and colour was conducted in the existence of 10,000 mg/L of SnCl_4,_ which was the optimum dosage pre-determined before and in the pH range of 3–11.

The ZP during the removal of SS and colour showed a gradual decrease throughout the pH values. Starting from pH3, the ZP increased slightly from 13.6 mV to 15.2 mV at pH5. Then, it slowly started to become less positive as the pH moved from pH6 until it approached a negative ZP value. The ZP values in the range of pH7 to pH8 were −1.8 mv to −0.4 mV, respectively, approaching the zero value. The point where the ZP becomes zero is known as the isoelectric point (IEP). Therefore, it can be said that the IEP value for SnCl_4_ to remove the pollutants from leachate lies in this pH range (pH7–8). Starting from pH7 onwards, the ZP of the sample became more negative, from −1.8 mV at pH7 to −22.4 mV at pH11.

This can be correlated from the results in Figure 3 showing that the removals were obtained at a pH range between 7 and 8 with an associated particle size of 2419 d·nm and ZP of −0.4 mV. This enhances the settlement of the flocs. The removal for colour and SS at this pH range was about 98% for both. The particle size at approaching zero ZP will have a greater size compared to the particles with a higher (more negative or positive) ZP, which occurred at acidic (pH < 6) and basic (pH > 8) conditions, as shown in Figure 5. This situation happens because the particle surface charge in the IEP range (pH7–8) is less compared to the more negatively or too positively charged ZP. The thickness of the diffusion layer in the particles is reduced when the surface charge of the particle is lower. The reduction in the thickness of the layer of particles hence will decrease the repulsive force between the particles. This led to the agglomeration and induced them to come closer and form a much larger particle [39,40].

Meanwhile, the effect of pH on the ZP and particle size in removing the SS and colour from the leachate using RS as the main coagulant is shown in Figure 6. Compared to ZP and particle size when SnCl_4_ was used as the coagulant, there was not much effect of RS as a coagulant on the ZP and the size of the particles. As the pH moved from pH3 to pH11, the ZP of the sample became more and more negatively charged. It started with −6.3 mV at pH3 to −19.7 mV at pH11. Since the ZP of the sample became more negatively charged, the size of the particle also became much smaller, which contributed to low removals for pollutants. The highest removal obtained at pH3 was 46.7% for SS and 76.5% for colour with an associated ZP of −6.3 mV and an average particle size of 933 d·nm. As the pH increased, the removal of SS and colour started to decrease. Smaller particle sizes were recorded with an increase in pH. For example, the particles at pH4 were 535 d·nm and reduced to 234 d·nm at pH11. These conditions did not favour the settlement of flocs. This is because the negatively charged particle (pH6–11) appear to repel each other and prevent the particles from coming closer to form much bigger particles.

### 3.5. Comparisons of SnCl_4_ with Different Types of Metal Coagulants

Table 2 compares the performance of different metal salts and their valencies in landfill leachate treatment.

Compared with the previous works, the raw leachate that we examined in the current study was generally the highest initial concentration of the raw leachate (colour 22,970 PtCo and SS 548 mg/L). A higher concentration of leachate pollutants usually requires a higher coagulant dosage. Our conclusion (8000 SnCl_4_ and 1000 RS) in the current work is considered in line with the results of previous workers, especially by Rui et al., (2015) [41] (9000 mg/L Alum). However, they used less concentrated leachate. Even though lower dosages of metal salts were found by other researchers, most of them used fairly low to moderate levels of leachate pollutants.

### 3.6. Operating Cost Calculation

The estimation of the coagulation treatment costs in removing suspended solids and colour removal is shown in Table 3. However, our calculation was derived from laboratory work only. The reagent cost involved the three types of chemical reagents: hydrochloric acid (HCl) and sodium hydroxide (NaOH) for pH alteration, and tin tetrachloride (SnCl_4_). The energy cost was estimated according to the electricity unit cost based on the electricity tariff by our local energy provider, i.e., Tenaga Nasional Berhad (TNB), Malaysia.

## 4. Conclusions

- At pH8 and with 10,000 mg/L concentration, SnCl_4_ as the main coagulant was able to remove 99% and 97.3% of SS and colour, respectively, with an associated particle size of 2850.7 d·nm and ZP values of −0.4 mV.
- RS was ineffective as a sole coagulant with only 46.7% and 76.5% reductions in SS and colour, respectively, at pH3, a 6000 mg/L dosage, 933 d·nm, and a ZP value of −6.3 mV.
- In the presence of 1000 mg/L of RS, the dosage of SnCl_4_ could be reduced from 10,000 mg/L to 8000 mg/L by the bridging mechanism, with almost complete removal of 100% of SS and colour.
- As a sole landfill leachate treatment system, this study concludes that RS showed better potential as a coagulant aid compared to when it is used as a main coagulant.

## Figures and Tables

**Table 1:** Raw compositions of Alor Pongsu Landfill leachate.

Parameter.	Min	Max	Average	Discharge Limit ^1^	Method Number	Instrument
pH			8.12	6.0–9.0		
BOD_5_ (mg/L)	45	112	85	20	5210 B	DO meter
COD (mg/L)	1390	5078	2937	400	5220 D	DR Spectrophotometer
BOD_5_/COD	0.02	0.07	0.03	-		
Suspended Solids (mg/L)	258	547	411	50	HACH Methd 8006	DR Spectrophotometer
Ammoniacal nitrogen (mg/L NH_3_-N)	1160	2820	1448	5	HACH Method 8038	DR Spectrophotometer
Colour (Pt.Co)	9480	22,970	15,062	-	HACH Method 8025	DR Spectrophotometer
Turbidity (NTU)	9.68	44.59	22.0		APHA 2130 B	Turbiditimeter
Zeta Potential	−18.6	−22.4	−20.5			Zetasizer

^1^ According to Environmental Quality (Control of Pollution from Solid Waste Transfer Station and Landfill) Regulations 2009 (PU (A) 433).

**Table 2:** Comparisons of optimal dosage on the removal of SS and colour using different types of coagulant from landfill leachate.

Types of Coagulant	Optimal Dosage (mg/L)	Optimal pH	Raw Leachate Concentration		Efficiency Removal (%)		References
			Colour (PtCo)	SS (mg/L)	Colour	SS	
Aluminium Sulfate	9000	7	4372	351	84	96	[41]
PAC	5000	6	5517.5	745	98	99.5	[25]
FeCl_3_	3000	7	4372	351	92	89	[41]
	3600	6	5318	297	95.5	-	[20]
	1500	6	3199	407	95	94	[42]
ZrCl_4_	1500	4	5000	441	94.3	93.4	
TiCl_4_	1200	4	17,075	397	82	92	[29]
	600	6	4253	330	81.4	86.7	[19]
SnCl_4_	10,000	8	22,970	548	97.3	99	This Study
SnCl_4_ + RS	8000 SnCl_4_ + 1000 RS	8	22,970	548	97.6	100	This Study

**Table 3:** The quantity and cost of coagulant required for SS and colour treatment at lab scale (per 3 L of leachate).

Item	Estimated Usage	Cost (per kg or L or W)	Total	Reference
Reagents Cost				
HCl MERK 37%	25 mL	RM 550/2.5 L	RM 5.50	[43,44]
NaOH 97% Pellets	25 mL	RM 335/500 g	RM 16.80	
SnCl_4_	75 mL (for 6 beakers)	RM 269/100 g	RM 201.8	
Rubber Seed	120 mL (for 6 beakers)	RM 1.00/kg	RM 120.00	
Energy Cost				
Jar test (VELP-Scientifica, Model: JLT6)	19 W	RM 0.73/kW	RM 14.00	[45]
		Total	RM 358.10	

## Data Availability

Not applicable.
